# Similarity-Based Interference and the Acquisition of Adjunct Control

**DOI:** 10.3389/fpsyg.2017.01822

**Published:** 2017-10-18

**Authors:** Juliana Gerard, Jeffrey Lidz, Shalom Zuckerman, Manuela Pinto

**Affiliations:** ^1^School of Communication and Media, Ulster University, Jordanstown, United Kingdom; ^2^Department of Linguistics, University of Maryland, College Park, MD, United States; ^3^Utrecht Institute of Linguistics OTS, Department of Languages, Literature and Communication, Utrecht University, Utrecht, Netherlands

**Keywords:** adjunct control, language acquisition, similarity-based interference, intervention, binding, anaphora

## Abstract

Previous research on the acquisition of adjunct control has observed non-adultlike behavior for sentences like “*John bumped Mary after tripping on the sidewalk*.” While adults only allow a subject control interpretation for these sentences (that John tripped on the sidewalk), preschool-aged children have been reported to allow a much wider range of interpretations. A number of different tasks have been used with the aim of identifying a grammatical source of children’s errors. In this paper, we consider the role of extragrammatical factors. In two comprehension experiments, we demonstrate that error rates go up when the similarity increases between an antecedent and a linearly intervening noun phrase, first with similarity in gender, and next with similarity in number marking. This suggests that difficulties with adjunct control are to be explained (at least in part) by the sentence processing mechanisms that underlie similarity-based interference in adults.

## Introduction

By age 4, children’s language abilities are already quite sophisticated, but there are still many ways in which they seem to differ linguistically from adults. Non-adultlike interpretations for a variety of structures have been widely reported in children age 4 and older; however, there is not always a consensus regarding the source of children’s behavior. Even less clear are the mechanisms involved in the transition from non-adultlike behavior to adultlike behavior. In this paper, we present a case study in accounting for errors in children’s interpretations in terms of sentence processing mechanisms, rather than their grammatical representations. This kind of account, in addition to providing a better explanation for children’s behavior, also allows for a more continuous developmental path to adultlike behavior.

Without direct access to what children know about their language, we are dependent on indirect behavioral measures that involve the interaction of linguistic knowledge with more general systems like those involved in memory and planning. Therefore, although non-adultlike behavior on a linguistic task may be due to immature linguistic knowledge, it may also result from this interaction with more general systems, despite adultlike knowledge. This paper investigates a case of linguistic dependencies in children that has received little attention recently, but which has no clear account to date. In sentences with adjunct control as in (1) below, the unpronounced subject of a temporal adjunct is bound by the main clause subject, but not the object. Here we annotate the missing subject as PRO, although the experiments in this paper do not depend on the precise syntactic representation of the control relation.

(1) John_1_ bumped Mary_2_ after PRO_1/^∗^2_ tripping on the sidewalk.

For sentences like (1), adults only allow a subject control interpretation [*John* in (1)], but 4–6 years old children have been reported to allow a range of non-adultlike interpretations, including object control (*Mary*), control by any sentence-internal referent (*John* or *Mary*) but not by a sentence-external referent, and free interpretation (**Table 1**).

**Table 1:** Reported non-adultlike patterns and accounts in previous studies.

	Reported non-adultlike pattern(s)			
		
Study	Object control	Sentence-internal	Free reference	Account
Goodluck (1981)	x	x	x	Variable Attachment
Hsu et al. (1985)	x	x	x	Variable Attachment
McDaniel et al. (1991)	x	x	x	Variable Attachment
Cairns et al. (1994)	x	x	x	Variable Attachment
Broihier and Wexler (1995)			x	Nominalization
Goodluck (2001)			x	Nominalization
Adler (2006)			x	Variable Attachment


Multiple factors may be involved in the patterns of behavior observed in previous studies on children’s acquisition of adjunct control: although children’s behavior might be due to non-adultlike knowledge, it may also be a result of task effects or the interaction of adultlike knowledge and non-adultlike general systems. Nevertheless, all accounts to date have assumed that children’s behavior is due to an immature grammar. Several accounts have proposed different non-adultlike grammars to account for the behavior observed in previous studies; however, no one proposal clearly provides a better explanation than the others because each accounts for only a subset of the observations in the literature. In this paper we consider the role of general cognitive systems in processing sentences like (1) in order to explain children’s behavior in previous studies. In two experiments, we show that children’s accuracy for sentences with adjunct control is modulated by similarity-based interference, suggesting that children deploy the same parsing procedures as adults, but are more susceptible to interference.

## Previous Grammar-Based Accounts

Two main grammar-based accounts –Variable Attachment and Nominalization – have been proposed to explain children’s non-adultlike behavior for sentences like (1) (**Table 1**).

Citing the generalization that PRO is bound by the closest c-commanding NP (Chomsky, 1981), those who proposed the Variable Attachment account have argued that non-adultlike behavior for (1) is due to incorrect attachment of the adjunct to the main clause (Goodluck, 1981; Hsu et al., 1985; McDaniel and Cairns, 1990; McDaniel et al., 1991; Cairns et al., 1994; Adler, 2006). Adultlike behavior results from attaching the adjunct to a position higher than the object so that only the subject can bind PRO, while non-adultlike behavior results from attaching the adjunct too low, such that both the subject and the object can bind PRO, or from attaching the adjunct too high, such that no main clause argument can bind PRO (resulting in a discourse-driven interpretation). The studies supporting the Variable Attachment hypothesis have been criticized, however, for categorizing children as having one non-adultlike grammar or another based on too few observations from any single child, and for arguing for distinct non-adultlike grammars based on small sample sizes for each grammar (Wexler, 1992; Broihier and Wexler, 1995). As an alternative account, Wexler (1992; Broihier and Wexler, 1995) and Goodluck (2001) argued for the Nominalization account – that all children who allowed any non-adultlike interpretation of (1) have the same non-adultlike structure – a nominal adjunct without PRO, similar to:

(2) John bumped Mary after the tripping on the sidewalk.

In contrast to (1), who tripped on the sidewalk in (2) is underspecified, and adult speakers report that (2) can mean that *John*, *Mary*, or any other salient referent in the discourse tripped on the sidewalk.

In contrast to the Variable Attachment account with three distinct non-adultlike states, Wexler (1992) argued that preschool-aged children either have a nominal representation similar to (2), that allows a free interpretation of PRO, or they have the adult grammar (strict subject control). Since the number of observations in previous studies was not sufficient to reliably identify children who exhibited some, but not all, of the reported non-adultlike interpretations, Wexler (1992) argued that these children likely would have exhibited a broader range of interpretations with a more exhaustive paradigm. Indeed, using a more extensive set of conditions, Broihier and Wexler (1995) found that preschool-aged children who were not adultlike did allow a free interpretation of PRO, consistent with the nominal structure in (2), with later support from Goodluck (2001).

Despite the arguments in support of the Nominalization account, a number of questions remain regarding the source of children’s behavior in previous studies. First, although a pattern of behavior with only two groups of children – one adultlike, and one non-adultlike – as reported in Broihier and Wexler (1995) is consistent with the nominal structure in (2), it is just as consistent with the high attachment structure proposed by the Variable Attachment account: specifically, both structures predict free interpretation of PRO. Thus, while Wexler’s (1992) argument against four *distinct* states as proposed in the Variable Attachment hypothesis is likely correct, Broihier and Wexler’s (1995) study does not provide conclusive evidence against incorrect attachment of the adjunct altogether.

Second, neither the Nominalization account nor the Variable Attachment account make predictions about the rate at which a non-adultlike interpretation should be accessed in place of the adultlike interpretation. While both the nominal structure in (2) and the high attachment structure from the Variable Attachment account predict the free interpretation of PRO, in all of the studies to date, children accessed different interpretations at different rates. Although this variation is not inconsistent with either account, no explanation is offered for why, for example, uniform rates for each interpretation of PRO were never observed – a result that would also have been consistent with these accounts. In sum, any pattern of behavior with non-zero rates of non-adultlike interpretations of (1) would be consistent with both the nominal and high attachment structures, since neither places a syntactic restriction on the interpretation of the adjunct subject.

To summarize previous research on the acquisition of adjunct control, a number of studies have reported that children exhibit non-adultlike behavior for sentences like (1), but at different rates, and for unclear reasons. Furthermore, no conclusive evidence has been provided regarding the source of children’s behavior. We propose that non-adultlike behavior in previous studies can be attributed to similarity-based interference effects, despite adultlike knowledge.

## Interference Effects in Adults

Similarity-based interference is observed when a grammatically inaccessible element matches in features with the grammatically accessible antecedent in a linguistic dependency. For example, interference has been reported for object relative clauses (Gordon et al., 2001, 2004; Warren and Gibson, 2002, 2005; Gordon et al., 2006; for a review see Gordon and Lowder, 2012):

(3) a. The banker that the lawyer admired ___ climbed the mountain.
  b. The banker that you/Joe admired ___ climbed the mountain.

In (3), there is a dependency between the head of the relative (*the banker*) and the object gap, after *admired*. In both (3a) and (3b), the relative clause subject intervenes between the head of the relative and the object gap, but in (3a) the head and the subject match in the feature NP type (both full NPs), while in (3b) the head (*the banker*) mismatches with the subject (*you* or *Joe*) in NP type. For relative clauses where the head and the subject match in features [as in (3a)], more interference is reported compared to when these features mismatch [as in (3b)]. Interference effects have been reported in adults in terms of reading or reaction times, and have been observed for several different types of linguistic dependencies [see Jäger et al. (2017) for a review].

Much of the work on similarity-based interference has focused on interference during retrieval of a target, based on specific cues for retrieval. For example, when linking a verb to its subject, the verb can be marked in English for number. In the ungrammatical case where the number feature on the subject does not match with the cue specified on the verb, but there is a grammatically inaccessible NP which does match the cue [as in (4)], illusions of grammaticality are observed, due to the grammatically inaccessible NP (Bock and Miller, 1991; Clifton et al., 1999; Pearlmutter et al., 1999; Eberhard et al., 2005; Häussler and Bader, 2009; for a review see Wagers et al., 2009).

(4) ^∗^[The key_SG_ [to the cabinets_PL_]] are_PL_ on the table.

In cases like (4) with explicit retrieval cues, reading time differences may be attributed to interference with the retrieval mechanism, by a grammatically inaccessible but matching intervener. The effects observed for object relative clauses in (3), however, suggest that similarity-based interference is not always dependent on retrieval cues, because NP type is not specified as a cue for retrieval [but see Van Dyke and McElree (2006) regarding the specificity of semantic properties of different NP types]. That is, while the form of the verb is marked for agreement with the number of the subject (and the form of a pronoun or reflexive specifies the number and gender of the antecedent), NP type is not specified on the verb in (3) in the same way that number is explicitly specified on the verb in (4). Instead, similarity-based interference in (3a) must arise from the initial encoding of the target and intervener, or while storing both elements in memory.

Research on similarity-based interference in language processing has discussed the effects primarily in terms of a storage account [but see Johnson et al. (2011)]. Meanwhile, extensive evidence is provided for interference during encoding the domain of visual processing (Treisman and Gormican, 1988; Lavie, 1995; Treisman, 1996; Luck et al., 1997; Luck and Ford, 1998; Luck and Vogel, 2001; Hopf et al., 2002; Alvarez and Franconeri, 2007; for a review, see Brady et al., 2011). With interference effects observed in other domains in addition to language, the question arises of how much of the general phenomenon – interference with similar items – is due to properties of domain general memory mechanisms, which then interface with domain specific systems. With effects of similarity-based interference observed for features like NP type, at least some aspects of interference in sentence processing must be domain specific, since values like “name” and “definite description” do not provide a meaningful distinction in other, non-linguistic domains. Analogously, interference in visual processing has been observed for objects that share features related to shape and orientation in space, i.e., features that are meaningful in the visual domain, but not, for example, in the linguistic domain.

One possibility is that these effects are completely unrelated, with no overlap between the memory mechanisms that are involved in encoding and storing linguistic representations and the mechanisms that are deployed in visual processing. An advantage of this is that it provides an intuitive way to explain the different ways that similarity-based interference is realized in different domains: in general, interference effects are observed for representations that match in features compared to ones that mismatch, but there are domain specific differences in how the effects are modulated by other factors (e.g., timing, additional items). A disadvantage of this model, though, is that it involves redundancy. Specifically, it requires multiple unrelated mechanisms to account for interference effects in different domains, which fails to capture the *general* observation that encoding and storing similar items results in interference.

An alternative approach appeals to general properties of the memory architecture to explain this observation – in particular, that the architecture is feature-driven (Nairne, 1988, 1990). Meanwhile, domain specific properties are responsible for the variation in interference effects across domains. As a consequence of the memory architecture, similar representations will interfere with each other in memory; however, the features that make up these representations will be largely domain specific. Furthermore, the kinds of features that determine similarity vary widely across domains – an expected source of variation, given the range in perceptual channels through which the representations are generated. Differences in the ways that interference effects are realized in different domains therefore result from differences in the specific properties of the representations that are stored in memory: interference effects in language processing, for example, are sensitive to linguistic features, while interference effects observed for visual processing are sensitive to constraints in the visual system.

With no definitive evidence for storage or encoding (or both) as the source of similarity-based interference in cases where there is no explicit cue for retrieval, both options may be considered when evaluating children’s knowledge of linguistic dependencies. While the paradigms used with children are much more varied than those used with adults, a general finding is that children exhibit lower accuracy in the same contexts that adults exhibit slowdowns in reading times (Costa et al., 2012; see Omaki and Lidz, 2015 for a review). The following section discusses the implications of these parallel effects for how children encode linguistic dependencies, including adjunct control.

## Interference Effects in Children

A number of studies on language acquisition in children have manipulated the feature match between a target and intervener (**Table 2**).

**Table 2:** Interference effects observed in previous studies with children.

Construction	Studies
Relative clauses	Kidd et al. (2007); Arnon (2009); Brandt et al. (2009); Friedmann et al. (2009); Adani et al. (2010, 2014); Belletti et al. (2012); Costa et al. (2012); Bentea and Durrleman-Tame (2013); Haendler et al. (2015), a.o.
Subject-to-subject raising	Choe and Deen (2015); Choe and O’Grady (2016)
Object fronting	Sauermann and Höhle (2016)
Non-reflexive pronouns	Clackson et al. (2011)


*The effects are realized as differences in accuracy, with lower accuracy observed when target and intervener match in features than when they mismatch.*

The majority of the studies in **Table 2** used a picture selection task, but a few of them used a Truth Value Judgment Task (Choe and Deen, 2015; Choe and O’Grady, 2016; Sauermann and Höhle, 2016) or a visual world paradigm (Clackson et al., 2011). In addition to NP type, researchers have varied the animacy (Kidd et al., 2007; Brandt et al., 2009; Bentea and Durrleman-Tame, 2013), the number (Adani et al., 2010, 2014), and the gender (Adani et al., 2010, 2014; Belletti et al., 2012) of the target and intervener.

The effects of NP type and animacy, which are not explicitly marked in any of the structures in **Table 2**, suggest that the interference effects observed for children have the same source as those observed for adults with clefts and relative clauses. However, conflicting results in previous experiments, as well as different assumptions about the source of children’s errors have resulted in several different perspectives regarding interference effects in children, as opposed to adults.

For example, while effects of NP type and animacy have consistently been observed without any explicit retrieval cues, interference effects for gender have only been observed when gender is explicitly marked on the verb, with no interference observed the verb is not marked for gender agreement (Adani et al., 2010, 2014; Belletti et al., 2012). While this difference is accounted for by appealing to the difference in the availability of gender cues, other task-related factors may have contributed to the lack of an effect when the verb was not marked for gender agreement (e.g., minimal context before the test sentence, item effects).

In addition to the role of the specific features, researchers have also disagreed about the source of the effects in the first place. While a few studies have considered a similarity-based interference account of children’s errors for linguistic dependencies (Choe and Deen, 2015; Choe and O’Grady, 2016), more commonly cited accounts are Child Relativized Minimality, which posits that children have a non-adultlike grammar that is overly restrictive in cases with overlapping features (Friedmann et al., 2009), and input accounts, which cite the frequencies of relative clauses in the input as determining which features will be preferred where (Kidd et al., 2007; Brandt et al., 2009).

These input accounts are generally based on instances of a particular type of structure in the input – for example, relative clauses; meanwhile, Relativized Minimality accounts for generalizations across a range of structures, based on hierarchical relations between constituents. In the adult grammar, Relativized Minimality disallows a dependency between two constituents when an intervener c-commands the lower constituent and overlaps completely with the higher constituent in features that trigger movement (Rizzi, 1990, 2004; Chomsky, 1995). This restriction is not specific to any one type of dependency, and also allows for a unified explanation for wh-island effects (5b) and *super-raising* (6b):

(5) a. What_+Q_ did you say John read what?
  b. ^∗^What_+Q_ did you say who_+Q_ read what?

(6) a. John seems John to be likely John to win.
  b. ^∗^John seems that it is likely John to win.

In (5a), there is an A-bar dependency between the final position of the wh-word *what* in the main clause and its initial position in the embedded clause. With no intervening elements in an A-bar position that overlap in features with *what* in (5a), there is no minimality violation. In contrast, the same dependency is disallowed in (5b), because the wh-word *who* intervenes in an A-bar position, and bears the same +Q feature as *what*.

Similarly, the same analysis is available for the contrast in (6), but with A-movement rather than A-bar movement: in (6a), the main clause subject *John* raises from an A-position in the most embedded clause, to an A-position in the intermediate clause, to an A-position in the main clause, without crossing any other constituents in an A-position. In contrast, the dependency in (6b) is ruled out by Relativized Minimality because the expletive *it* intervenes in an A-position, and is of the same type as *John*.

Importantly, Relativized Minimality is stated purely in hierarchical terms, without referring to linear order. For example, a dependency is blocked in (5b) with the intervener *who* that both precedes *and* c-commands the initial position of *what* in the embedded clause. However, there is no minimality violation with no c-command relation, even with the same linear precedence relation:

(7) What_+Q_ did you say [the boy who_+Q_ left] read what?

In contrast with (5b), *who* in (7) does not c-command the initial position of *what*. As a result, *who* does not block the dependency, despite being a linear intervener.

While the adult grammar only prohibits sentences with complete overlap in the relevant features, the non-adultlike grammar under a Relativized Minimality account is much more restrictive. While children do exhibit sensitivity to overlap in +Q feature in (5), the non-adultlike grammar also disallows structures with partial overlap, which are not ruled out by the adult grammar. This account explains children’s poor performance with object relative clauses like (3a), for example, because *the banker* and *the lawyer* overlap in NP type, even though only one bears the +Q feature.

(3) a. The banker that the lawyer admired ___ climbed the mountain.
  b. The banker that you/Joe admired ___ climbed the mountain.

Child Relativized Minimality has been proposed as a source of non-adultlike behavior for various dependencies; importantly, for all of these there was a c-command relation between the elements in the dependency, *as well as* the intervener. For example, in (3), the relative clause head c-commands the object gap and the intervening subject (*the lawyer/you/Joe*), and the intervening subject c-commands the object gap.

However, the focus of the current study is on children’s non-adultlike behavior for sentences with adjunct control, repeated below in (1) – a structure which is not consistent with hierarchical intervention. In sentences with adjunct control, the antecedent of adjunct PRO is the *closest* c-commanding NP, and an NP object intervenes linearly, but not hierarchically, between the main clause subject and adjunct PRO.

(1) John_1_ bumped Mary_2_ after PRO_1/^∗^2_ tripping on the sidewalk.

Thus, if children exhibit interference effects for sentences with adjunct control, the effects cannot be due to hierarchical intervention.

In contexts where interference effects are observed, where no c-command relation is possible between an intervener and the elements in the dependency (Franck et al., 2006; Friedmann and Costa, 2010; Clackson et al., 2011), one possibility is that the same interference effects in adults are also realized in children, with more interference predicted for children than for adults. If so, the same questions arise for children about similarity-based interference based on storage and encoding in memory, but also about how to account for differences between children and adults.

For example, as mentioned in §3, similarity-based interference in adults may occur while matching items are stored in memory prior to retrieval, but also during encoding when an item matches in features with another item that is already stored in memory. If similarity-based interference is responsible for interference effects in children as well as in adults, then the effects in children are predicted to be amplified compared to adults. Given that executive function, including the ability to access and manipulate information in memory, is slower to develop overall, different explanations are available for the greater interference effects under storage compared to encoding accounts (Courage and Cowan, 2008; Mazuka et al., 2009; Novick et al., 2010; Omaki and Lidz, 2015).

More interference in match conditions is predicted under a storage account, for example, if the representations of the target and intervener in memory are quicker to decay over time in children than in adults (Courage and Cowan, 2008). If the features distinguishing the target and intervener become less accessible over time, then a higher rate of misretrieval is predicted in contexts where fewer features distinguish the target from the intervener (i.e., match contexts). In contrast, with more features available to discriminate between the target and the intervener in mismatch contexts, differences between the target and intervener will take longer to decay. As a result, misretrieval is predicted to be less likely in a mismatch context at the same point in time as for a match context.

Under an encoding account, more interference in match conditions is predicted if children are less competent than adults at encoding the target and intervener as sufficiently distinct in memory. When fewer features distinguish the target from the intervener (in a match context), they are less likely to be encoded as distinct compared to when more features are available to distinguish the target from the intervener (in a mismatch context). Additionally, items that are encoded as less distinct from each other may be more susceptible to feature displacement for features that are distinctive (Jäger et al., 2017), increasing the likelihood that an incorrect item will be retrieved in match contexts, compared to mismatch contexts.

While the encoding and storage accounts provide different explanations for children’s non-adultlike behavior, both accounts predict a higher rate of non-adultlike interpretations in match contexts (i.e., when the target and intervener match in features) than in mismatch contexts. Furthermore, under both accounts, non-adultlike interpretations arise from retrieving the intervener instead of the target (misretrieval). For object relative clauses like (3a), the adultlike interpretation is to link the object gap with the head of the relative clause; meanwhile, retrieving the intervener instead of the target would cause the object gap to be linked to the relative clause subject, rather than the head of the relative:

(8) The banker that the lawyer admired the lawyer climbed the mountain.

The resulting interpretation is a reading in which the lawyer would have both the subject and the object θ-roles. While this interpretation is predicted in cases of misretrieval for object relative clauses, it has not typically been included as an option in previous studies on the acquisition of relative clauses. Many of the studies have used a picture selection task, with a choice between, e.g., a lawyer admiring a banker and a banker admiring a lawyer. Since both choices are only a partial match for the interpretation in (8) (i.e., one picture has the lawyer as the agent and other has the lawyer as patient), chance performance would be predicted when the object gap is linked to the intervener rather than the target.

While the nature of children’s final interpretations for object relative clauses after misretrieval is not entirely clear, other structures for which interference effects have been demonstrated allow for more straightforward predictions. For example, children have been reported to exhibit non-adultlike interpretations for sentences with raising from the subject position of the embedded clause to the subject position of the main clause (9):

(9) John seems to Mary [John to be happy].

In (9), the subject of the embedded clause is not pronounced, but must be linked to the subject of the main clause to receive an interpretation. However, children have been reported to show chance interpretation for the embedded subject in (9) between an adultlike interpretation and an interpretation with the intervening experiencer (*Mary*) as the antecedent of the embedded subject. This non-adultlike interpretation is predicted by similarity-based interference (Choe and Deen, 2015; Choe and O’Grady, 2016), where the intervening experiencer (*Mary*) is more likely to be retrieved in place of the target (*John*) when they interfere with each other due to feature overlap, compared to when they do not overlap in features. Indeed, Choe and Deen (2015) showed that children were less likely to retrieve the intervening experiencer when the target and intervener mismatched in NP type than when they matched.

Like the subject raising dependency in (9), sentences with adjunct control (1) also involve a syntactic dependency between the main clause subject (*John*) and an unpronounced subject in a separate clause:

(1) John_1_ bumped Mary_2_ after PRO_1/^∗^2_ tripping on the sidewalk.

Additionally, both sentences have an intervener (*Mary*), such that retrieving the intervener rather than the target results in a specific non-adultlike interpretation of the unpronounced subject.

Meanwhile, unlike sentences with subject raising (in which the antecedent is specified as a feature of the selectional criteria for the main clause verb), the antecedent of adjunct PRO is determined based on the structure of the sentence: in relative clauses, the target is the head of the relative clause, while in sentences with adjunct control, the target is the main clause subject.

One source of debate about interference effects concerns the role of agreement, which may be used as a cue for retrieval for some features (gender, number), but not for others (animacy, NP type). While the effects observed for animacy and NP type suggest that similarity-based interference occurs independent of retrieval in both children and adults, the results for gender and number are less clear.

Sentences with adjunct control, with a non-finite verb that is not marked for agreement, present the opportunity to investigate similarity-based interference in a context without any explicit retrieval cues. One exception is that interference effects for animacy have been observed for sentences with adjunct control in adults, with the argument that animacy may be used as a retrieval cue despite the lack of any explicit agreement marking (Parker et al., 2015). Animate antecedents were also preferred over inanimate ones; this may be due to specific verbs used in the test sentences, if they were more likely to occur with an animate subject. However, another option is that a learned association is developed for the structurally defined antecedent of adjunct PRO, due to the higher probability of an animate subject over an inanimate on [i.e., cue confusion from (Jäger et al., 2017)].

If children’s non-adultlike interpretations arise from similarity-based interference between the target and the intervener in encoding or in storage, then the same interference effects as observed for relative clauses should be found for adjunct control. If so, then children’s non-adultlike behavior in previous studies on the acquisition of adjunct control may be attributed to similarity-based interference.

In the following sections, we present two experiments that manipulate the feature match between the target and intervener for sentences with adjunct control. In both experiments, children exhibit higher accuracy when the target and intervener mismatch in features than when they match. These results suggest that for sentences with adjunct control, the same parsing procedures that result in interference in adults are also deployed by children, and that children differ from adults in the resources at their disposal to deploy these procedures.

## Experiment 1: Gender Manipulation

Experiment 1 investigated whether interference would be observed when the target and intervener matched in gender. Under an interference account, items that overlap in gender should be more similar, and therefore more likely to interfere with each other prior to retrieval than items that are distinguished by gender, with all other features equal.

Additionally, in previous studies on the acquisition of adjunct control, the main clause subject and object in many of the test sentences overlapped in gender. If interference effects are observed in Experiment 1, then this overlap may have played a role in the observed non-adultlike behavior, independent of children’s grammars.

### Participants

Participants were 24 children (7 males) ages 3;11–5;3 (*M* = 4;8.6) who were recruited through the University of Maryland Infant and Child Studies Database or participated at their local preschools in the Washington, D.C. area. An additional six children were excluded from the final sample for answering too many control sentences incorrectly (5) or failure to complete the task (1).

Adult controls (*n* = 6) were also tested. They performed at 100% accuracy for all items with no variation, and their results are not included in further analyses. The adults were undergraduate students in introductory Linguistics classes at the University of Maryland, College Park, and they received course credit for their participation. This study was carried out in accordance with the recommendations of the Institutional Review Board at the University of Maryland, College Park with written informed consent from adult participants and the parents of child participants. All subjects gave written informed consent in accordance with the Declaration of Helsinki. The protocol was approved by the Institutional Review Board at the University of Maryland, College Park.

### Methods

**FIGURE 1:**
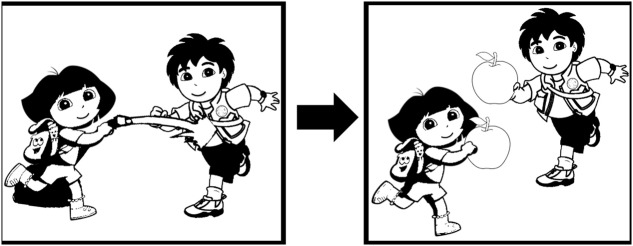
Example item for Experiment 1, to go with (12).

We used the Coloring Book task designed by Pinto and Zuckerman (2015; Zuckerman et al., 2016), which prompts children to reveal their interpretation of a test sentence by coloring in a black and white picture. Children were presented with static pictures (**Figure 1**), along with a description of the actions in the pictures that was delivered by the experimenter:

(10) In this picture we have Dora washing Diego, and then there’s Diego eating an apple, and there’s Dora eating an apple too.^1^

Next, a preamble was introduced by the experimenter (11) to balance the salience of each character before the experimenter delivered the test sentence (12):

(11) So here’s how we should color this picture of Dora and Diego.
(12) Dora washed Diego before PRO eating the red apple.

For all items, the main clause event was depicted in one picture (Dora washing Diego), while the other picture contained both characters performing the action described in the adjunct clause (eating an apple). Coloring in one of the two objects (Dora or Diego’s apple in **Figure 1**) corresponded to an adultlike interpretation of PRO [Dora in (12)], while the other object corresponded to a non-adultlike interpretation [Diego in (12)], with the correct antecedent of PRO (*Dora* or *Diego*) counterbalanced across items. Because interpreting the responses depended on children coloring only one of the objects, the task was administered on a touchscreen computer and programmed so that only the two relevant objects could be colored in, with just one tap needed to select a color and one tap to color an object. Additionally, children learned during the training session that only one object should be colored in for each trial.

To determine the effect of interference for gender, the gender feature was manipulated on the main clause subject and on the main clause object, allowing for a balanced manipulation of FEATURE MATCH (MATCH/MISMATCH) as a within-subjects factor. In addition to items like (12), where the target and intervener MISMATCH in gender, items were also included that contained Mickey Mouse, allowing for test items where both the subject and the object of the main clause were male (13).

(13) Mickey_MALE_ washed Diego_MALE_ before PRO eating the red apple.

To make sure that children’s interpretations were due to the adjunct control dependency, we included control sentences with a finite adjunct that had an overt pronoun subject, as in (14):

(14) a. Dora washed Diego before she ate the red apple. (pronoun subject antecedent)
  b. Dora washed Diego before he ate the red apple. (pronoun object antecedent)

High performance on the control items serves as an indication that both the subject antecedent interpretation [in (14a)] and the object antecedent interpretation [in (14b)] are available without a syntactic restriction. Adultlike behavior on the test sentences, then, can be interpreted as a preference that is specific to sentences with adjunct control, despite the availability of both interpretations in sentences with no control dependency. To balance the number of times each character appeared throughout the experiment, control items also included sentences with Mickey and Dora. This resulted in three training items, four gender MATCH test items with Mickey and Diego, four gender MISMATCH test items with Dora and Diego, four control items with Dora and Mickey, and four control items with Dora and Diego. Control items alternated with test items, and no items were included with Diego and Mickey with an overt pronoun; these would have been syntactically ambiguous, and might have influenced children’s interpretations on the unambiguous items.

All of the items were counterbalanced for antecedent and screen position across items and lists, and children who responded incorrectly to more than one control item with a subject pronoun *or* to more than one item with an object pronoun were excluded from the analysis. Test sentences all had the structure like in (12) and (13) with the complementizer *before* or *after*, with emphasis on the color, and the stimuli were presented to children with the Coloring Book app (Pinto and Zuckerman, 2015) on a Dell touchscreen PC. Each participant was tested in a single session that lasted from 10 to 15 min for the children, and less than 5 min for the adults.

### Results

**FIGURE 2:**
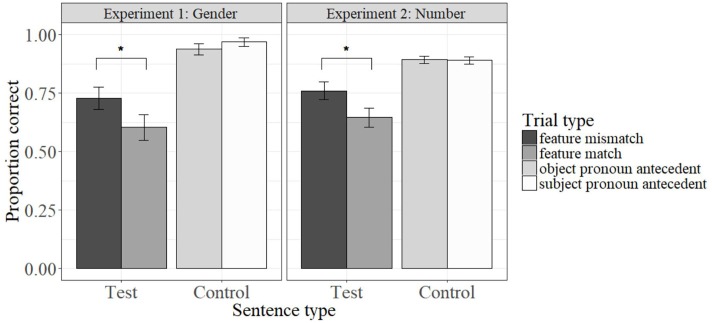
Results from Experiments 1 and 2, with proportion of correct answers for test sentences (MATCH/MISMATCH) in (12/13) and (15/16), and for control sentences in (14). ^∗^Means that there is a significant difference between the two conditions at *p* < 0.05.

Results for Experiments 1 and 2 are presented in **Figure 2**. For Experiment 1, we used R (R Core Team, 2015) and *lme4* (Bates et al., 2015) to perform a mixed-effects logistic regression analysis. The dependent variable was the proportion of ADULTLIKE responses. We entered subjects and items as random effects, and FEATURE MATCH as a fixed effect. A likelihood ratio test confirmed that the model with FEATURE MATCH outperformed the null model that included only random effects [χ^2^(1) = 3.96, *p* = 0.047], suggesting that FEATURE MATCH was a significant predictor for children’s performance. Since the dependent variable depended on the gender of the characters, we also confirmed that there was no further advantage for a model with FEATURE MATCH and participant gender as fixed effects [χ^2^(1) = 0.18, *p* = 0.67].

The fitted model revealed a main effect of FEATURE MATCH (β = -0.67, *Z* = -1.98, *p* = 0.048), with a *higher* proportion of ADULTLIKE responses in the MISMATCH condition (0.73) than for the MATCH condition (0.60).

Consistent with the predictions of a similarity-based interference account, children exhibited greater accuracy when the target and intervener mismatched in gender (12) than when they matched in gender (13). Furthermore, while accuracy in the MISMATCH condition was significantly higher than chance [two-tailed one sample *t*-test, *t*(23) = 4.84, *p* < 0.001], accuracy in the MATCH condition was only marginally higher than chance [two-tailed one sample *t*-test, *t*(23) = 1.93, *p* = 0.067].

### Discussion

In Experiment 1, children’s performance was significantly more accurate when the target and intervener mismatched in gender than when they matched in gender. This pattern of results is consistent with a similarity-based interference account, where children’s non-adultlike interpretations result from a failure to retrieve the correct antecedent. With shared features between the target and intervener in both conditions (e.g., number, animacy, and NP type), non-adultlike interpretations are still observed in MISMATCH contexts as well as MATCH contexts. However, with one *less* feature available to distinguish between the target and the intervener, the likelihood of a retrieval failure is greater in MATCH contexts than in MISMATCH contexts.

With a reliable effect of gender interference for sentences with adjunct control, the question arises of whether interference is observed only for gender, or for other features as well. If interference effects are observed only for gender, then this is problematic for an account of similarity-based interference. Since the account predicts that interference should be observed based on the similarity of the target and the intervener, no particular advantage is predicted for gender *per se*, especially when the dependency itself is not sensitive to the gender of the antecedent – as is the case for adjunct control. Therefore, it is important to confirm these results with other features to demonstrate that the interference effects observed in Experiment 1 are due to similarity-based interference, rather than a specific aspect of the gender feature.

In Experiment 2, the same feature match manipulation with sentences with adjunct control is repeated for number. As with gender, we find that children exhibit higher accuracy when the target and intervener mismatch in number than when they match, supporting the evidence from Experiment 1 for similarity-based interference as a source for children’s errors with adjunct control.

## Experiment 2: Number Manipulation

If the differences in accuracy observed in Experiment 1 were due to similarity-based interference, then the same differences between the MATCH and MISMATCH conditions are also predicted for other features, including number. As such, the same factors at play in Experiment 1 are also relevant for Experiment 2: if encoding and storing two singular NPs in memory raises the likelihood of retrieval failure by virtue of the two NPs sharing a number feature, then higher accuracy should be observed when the number feature is not shared (in the MISMATCH condition). Otherwise, if interference is only observed for number with an explicit retrieval cue, then no difference should be observed between the MATCH and MISMATCH conditions (since the adjunct verb is not marked for number agreement).

### Participants

Participants were 48 children (20 males) ages 4;0–5;5 (*M* = 4;10.28) who were recruited through the University of Maryland Infant and Child Studies Database or participated at their local preschools. An additional 20 children were excluded from the final sample for answering too many control sentences incorrectly (18), equipment failure (1), or a speech delay (1).

Adult controls (*n* = 4) were also tested, and performed at 100% accuracy for all of the test items with no variation. The adults were undergraduate students in introductory Linguistics classes at the University of Maryland, College Park, and they received course credit for their participation.

### Design, Materials, and Procedure

The design for Experiment 2 was largely the same as in Experiment 1, with a few key modifications. The modifications were made to allow for a manipulation of the number on the main clause subject and object in the test sentences with adjunct control, as well as in the control sentences with an overt pronoun.

First, to manipulate the number of the target and the intervener while still keeping word length as even as possible across conditions, the characters in Experiment 2 were two generic girls and two generic boys. This change allowed for a straightforward manipulation of the number on the main clause subject and object. As in Experiment 1, the independent variable was FEATURE MATCH (MATCH/MISMATCH), but with a singular target and intervener for the MATCH items (15), and a plural target *or* intervener for the MISMATCH items (16):

(15) The girl_SINGULAR_ washed the boy_SINGULAR_ before PRO eating the red apple.
(16) The girl_SINGULAR_ washed the boys_PLURAL_ before PRO eating the red apple.

**FIGURE 3:**
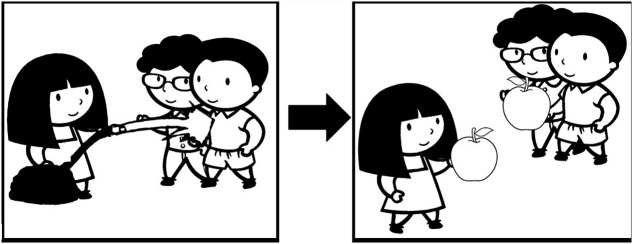
Example MISMATCH item for Experiment 2, to go with (16).

Items had the same form as in Experiment 1, but with two characters performing a single action when the test sentence included a plural NP (**Figure 3**).

Next, in contrast with Experiment 1, the manipulation of FEATURE MATCH in Experiment 2 was designed to be between-subjects, due to the number of items needed to fully counterbalance the roles across all four characters. As a result, each child saw the same number of test and control items as in Experiment 1; however, instead of seeing four test items in each condition, children were assigned to the MATCH condition or the MISMATCH condition, and saw test items only from their assigned condition. Assignment of children to conditions was random, except to balance the age ranges in each condition. Finally, half of the eight control items used two singular NPs (*the girl washed the boy…*), while the other half used one singular and one plural NP (*the girl washed the boys…*). This design is schematized in **Table 3**, with the items with a plural NP shaded in gray.

**Table 3:** Lists in Experiment 2.

MISMATCH test items	MATCH test items	Control items (same for both lists)
(1) Girls verb boy after…	Girl verb boy after…	Girl verb boy after she… (subject)
(2) Girl verb boys after…	Girl verb boy after…	Girl verb boys after they… (object)
(3) Girls verb boy before…	Girl verb boy before…	Girls verb boy before they… (subject)
(4) Girl verb boys before…	Girl verb boy before…	Girl verb boy before he… (object)
(5) Boy verb girls after…	Boy verb girl after…	Boy verb girls after he… (subject)
(6) Boys verb girl after…	Boy verb girl after…	Boy verb girl after she… (object)
(7) Boys verb girl before…	Boy verb girl before…	Boys verb girl before she… (object)
(8) Boy verb girls before…	Boy verb girl before…	Boy verb girl before he… (subject)

*The control items were the same in MISMATCH and MATCH conditions, and additional lists were created in both conditions to counterbalance the pronoun antecedent in the control items. Items with a plural NP are shaded grey.*

### Results and Discussion

As in Experiment 1, we used R (R Core Team, 2015) and *lme4* (Bates et al., 2015) to perform a mixed-effects logistic regression analysis of the relationship between the proportion of ADULTLIKE responses and the independent variable, FEATURE MATCH. We entered subjects and items into the model as random effects, with FEATURE MATCH as a fixed effect. A likelihood ratio test confirmed that the model with FEATURE MATCH outperformed the null model that included only random effects [χ^2^(1) = 4.38, *p* = 0.036], suggesting that FEATURE MATCH was a significant predictor for the proportion of ADULTLIKE responses.

The fitted model revealed a main effect of FEATURE MATCH (β = -0.58, *Z* = -2.12, *p* = 0.034), with a *higher* proportion of ADULTLIKE responses in the MISMATCH condition (0.76) than in the MATCH condition (0.64).

As in Experiment 1, and consistent with the predictions of a similarity-based interference account, children were more accurate in the MISMATCH condition than in the MATCH condition. Unlike in Experiment 1, two-tailed *t*-tests revealed that children’s accuracy was significantly greater than chance in *both* conditions, rather than just the MISMATCH condition [MISMATCH: *t*(23) = 7.08, *p* < 0.001; MATCH: *t*(23) = 3.62, *p* = 0.001]. However, this difference is expected, given that the target and intervener in Experiment 1 overlapped in both gender *and* number; meanwhile, the MATCH condition in Experiment 2 (with *the girl* and *the boy*) was more comparable to the MISMATCH condition in Experiment 1 (with *Dora* and *Diego*), since the target and intervener in both overlapped in number, but not in gender. The difference in accuracy between the MATCH condition in Experiment 2 (0.64) and the MISMATCH condition in Experiment 1 (0.73) is therefore unexpected, since they differ only in the NP type of the target and intervener. The implications of this difference will be explored further in the following section.

## General Discussion

Experiments 1 and 2 tested the prediction that similarity-based interference plays a role in predicting children’s non-adultlike interpretations of adjunct control. Previous studies have produced mixed results regarding the role of explicit cues in predicting interference effects. Furthermore, for features that are often realized with explicit agreement marking like gender and number, interference effects have only been observed in contexts where these features are in fact marked for agreement. Nevertheless, reliable effects were observed in both experiments in the present study for gender and number, despite the lack of any explicit retrieval cues on the adjunct verb. This suggests that children’s errors in previous studies on the acquisition of adjunct control were likely due at least in part to interference from the intervening object.^2^

From the results from Experiments 1 and 2, various questions arise about how children represent linguistic dependencies, from encoding to retrieval, and about additional sources of non-adultlike behavior. These questions are addressed in the following sections.

### How Would the Grammatically Inaccessible Antecedent be Retrieved When it Contrasts with the Target on the Relevant Structural Features?

In adults, interference effects are realized as slowdowns in reading time or reduced accuracy in comprehension questions. These measures may indicate temporary consideration of an ungrammatical antecedent; at the same time, studies with adults are not consistently designed to probe whether the grammatical antecedent was ultimately retrieved, despite the consideration of a matching distractor (Van Dyke, 2007; Patson and Husband, 2016; Schleuter et al., 2017). This raises the possibility that consideration of the ungrammatical antecedent might even lead to an ungrammatical interpretation even in adults. However, when the grammatical antecedent is distinguished from other partially matching items (e.g., main clause subject, for sentences with adjunct control) by structural cues alone, with no additional retrieval cues (e.g., number agreement), a cue-based retrieval mechanism should not be expected to retrieve an ungrammatical antecedent that does not match the structural cues (for sentences with adjunct control: any non-subject distractor).

Importantly, this depends on the availability of the relevant structural features upon deployment of the retrieval mechanism: to retrieve the main clause subject, the representation of the subject in memory must still be tagged as the subject, in contrast with the representations of non-subject elements. Meanwhile, if structural information decays over time, then this raises the possibility that at some point, the grammatical antecedent may no longer bear the relevant structural features. If so, then structural cues for retrieval will be less effective as more time elapses between encountering the target and deploying the retrieval mechanism.

In adults, structural information has been shown to decay much more quickly than semantic information. In studies testing recall of structural and semantic properties of sentences, accuracy rates are high for both types of properties immediately after a test sentence is presented. However, after a delay, both for sentence recall and for change detection, accuracy rates are much higher for a sentence’s meaning than for its particular structure, including information about the subject (Mehler, 1963; Sachs, 1967; Jarvella, 1971).

Although the reduced accuracy for structural information is observed in adults after a number of sentences, individual differences in recall accuracy are also observed (Gernsbacher, 1990). If these differences are related to differences in domain general memory processes – e.g., differences in overall memory capacity or decay rate – then we would predict that structural information will decay much more quickly in children than in adults. Thus, at high decay rates, the structural information about the main clause may no longer be available by the time that the retrieval mechanism is deployed in the adjunct clause, especially if children are less competent than adults at encoding the structural information in the first place.

Individual differences are also observed for interference effects themselves based on the strength of lexical representations, which may influence the quality of the representation that is encoded in memory (for a review see Van Dyke and Johns, 2012). If the same mechanisms are at play in children, then we also expect variation in interference effects, as lexical representations develop.

### For Which Other Structures do Children Exhibit Interference Effects, and Does This Interference Influence Their Acquisition?

An important implication of the interference effects observed in Experiments 1 and 2 is that, if these effects are indeed due to processes involved in encoding and storing elements in linguistic dependencies, then similar effects should also be observed for other types of linguistic dependencies that involve the same processes. Meanwhile, although feature match has been manipulated in a number of structures in studies with children (**Table 2**), there is a lot of variation across studies, and in some cases effects are observed for older children (i.e., 7 years and older) but not for younger children (Adani et al., 2010; Adani, 2011; Bentea et al., 2016; Bentea and Durrleman, 2017). In many of these contexts, however, the youngest children exhibited chance performance across the board, raising the possibility that the absence of any observed interference effects have also been related to the context of the task. The recent development of the coloring task (Pinto and Zuckerman, 2015), may therefore present an opportunity to revisit these structures in a more simplified context.

Furthermore, depending on what type of linguistic information is needed to acquire different types of dependencies, interference effects are predicted to influence how these dependencies are represented in the linguistic intake (as opposed to the input). Crucially, if similarity-based interference causes children to retrieve the wrong antecedent some proportion of the time, then this will directly affect the amount of noise in the *intake* (Gagliardi and Lidz, 2014; Omaki and Lidz, 2015), and may cause children to draw the wrong conclusions about their language, even with little noise in the *input*. If a significant proportion if the input is interpreted incorrectly due to similarity-based interference, then this will place much greater restrictions on what kinds of accounts are available for explaining children’s non-adultlike behavior. That is, any account that relies on children acquiring the adult grammar for a linguistic dependency by observing the relevant structure in the input must also consider how likely children would be to draw the wrong conclusions, due to noise in the *intake*. If similarity-based interference influences children’s interpretations in a high proportion of contexts, then accounts which appeal to distributional learning for syntactic development may face a significant challenge in accounting for this noise.

### Which Features Are Relevant for Similarity-Based Interference in Language?

The experiments presented in the present study found that children show similarity-based interference effects for gender and number, even when these features are not realized as explicit retrieval cues. This result is consistent with the effects observed in other studies that were also modulated by linguistic features; however, there were other features in addition to gender and number that differentiated the characters in the experiments from each other, particularly in Experiment 1. For example, the MISMATCH condition in Experiment 1 included Dora and Diego (who mismatch in gender), while the MATCH condition included Mickey and Diego (who match in gender). Although the control items also included pictures with Mickey and Dora to more evenly balance the combinations of characters throughout the experiment, other possible categorizations might be made based on, e.g., species (human vs. non-human), which would generate a different set of predictions than the predictions for gender: while Dora and Diego mismatch in gender, they match in species, and vice versa for Diego and Mickey. Similarly, the characters could also be categorized based on the fictional worlds that they appear in: Dora and Diego appear in the same world, whereas Mickey appears in a different one.

For both of these alternative categorizations, which categorize Dora and Diego together rather than Mickey and Diego, the opposite prediction would be made with respect to the interference effects. In Experiment 1, lower accuracy was observed in the MATCH condition which categorized Mickey and Diego together, based on gender. These results are therefore not consistent with these alternative categorizations as the relevant features for similarity-based interference. However, the results themselves do not provide an answer for *why* gender should be a better predictor of interference effects than other features like species or fictional world.

To address this question, the results of Experiment 1 and 2 must be considered in the context of other studies on similarity-based interference in language. In general, effects are observed for features that relevant for *linguistic* computation – i.e., that are realized as grammatical features in a language, even if the feature is not a retrieval cue for every dependency. Furthermore, a different profile is observed for features that encode semantic similarity (like a similarity in species) with no corresponding grammatical features (Lowder and Gordon, 2014; but see Van Dyke and McElree, 2006).

If interference effects in language arise as a result of overlap in *grammatical* features, however, the results of Experiments 1 and 2 raise some additional questions about the particular source of the effects.

First, in both the MISMATCH condition in Experiment 1 (12) and the MATCH condition in Experiment 2 (15), target and intervener overlapped in number (and NP type and animacy) but not in gender.

(12) Dora_FEMALE/SG_ washed Diego_MALE/SG_ before PRO eating the red apple.
(15) The girl_FEMALE/SG_ washed the boy_MALE/SG_ before PRO eating the red apple.

However, the accuracy for the MISMATCH condition in Experiment 1 (12) was nearly 10 percentage points higher than for the MATCH condition in Experiment 2 (15).

Additionally, there was essentially no difference between the MISMATCH condition in Experiment 2 – where the target and intervener mismatched in gender and number (*the girl_*FEMALE/SG*_ washed the boys_*MALE/PL*_…*) – and the MISMATCH condition in Experiment 1, with overlapping number (*Dora_*FEMALE/SG*_ washed Diego_*MALE/SG*_…*).

One source of the lower accuracy in Experiment 2 may be the differences between the two experiment designs, or differences in subject populations. At the same time, it is also worth considering how the differences between the gender and number features might give rise to different levels of interference, as well as the differences between the NP types used in the different experiments (i.e., the names used in Experiment 1 compared to the full NPs used in Experiment 2).

For example, nouns in English are always specified for number (either by the presence or absence of number agreement), and verbs are sometimes marked for number agreement. With some exceptions like “group,” the grammatical number marking on a noun agrees with its notional number. That is, *the girl* is grammatically singular and triggers singular agreement on a verb, and also refers to a single girl; similarly, *the girls* is grammatically plural and triggers plural agreement, and also refers to multiple girls. Meanwhile, words like “group” are exceptions, because they may be interpreted as singular or plural, depending on the context (Bock et al., 1999; Eberhard, 1999; Eberhard et al., 2005; Humphreys and Bock, 2005). This type of exception highlights the difference between the conceptual number of the referent (i.e., whether the NP refers to one or two girls) and the form of the referent (whether the NP is grammatically singular or plural). For number in English, these two properties usually align with each other.

From the design in Experiment 2 alone, it is not possible to distinguish between interference due to the storing two forms with the same grammatical number vs. interference due to representing two referents in memory with the same notional number. Distinguishing between these two possibilities can have implications for the variation in interference effects across languages, with wide variation in the extent to which different languages require explicit number agreement. For example, English has a much more impoverished system of number agreement than many other languages, which has been argued to influence English speaker’s interpretation of notional number, compared to speakers of languages with richer inflectional morphology (Vigliocco et al., 1995, 1996a,b). This would predict that interference effects for number in any given language depend on the number inflection in that language. However, other studies have found that English speakers exhibit increased sensitivity to notional number depending on its salience in a given context (Bock et al., 1999; Eberhard, 1999; Eberhard et al., 2005; Humphreys and Bock, 2005). This suggests instead that the presence of number marking in the form might have less of an influence on the conceptual representation, and that interference effects are more dependent on the similarity between referents, rather than forms.

Conceptual gender, unlike number, is only available for a few items (e.g., *man, woman*) but languages vary widely on the extent to which they make use of grammatical gender – from languages like Turkish and Mandarin Chinese, with no spoken gender marking, to languages like Spanish and German with grammatical gender on all nouns but no gender agreement on the verb, to languages like Hebrew and Russian, which also have verbal agreement for gender. In English, only pronouns and reflexives are grammatically marked for gender, and gender is not marked for agreement on the verb. As such, the overlap in gender in Experiment 1 was an overlap in conceptual rather than in grammatical gender (since *Dora*, *Diego*, and *Mickey* do not bear grammatical gender marking). This contrasts with the overlap in Experiment 2, which was grammatical as well as conceptual. The finding that children exhibited interference effects for gender as well as number supports an account where both effects are due to similarity in conceptual representations, rather than (or in addition to) form (Vigliocco and Franck, 1999, 2001; but see Lago et al., 2017); however, this does not account for the difference in accuracy between (12) and (15). Furthermore, since English does make use of gender agreement in pronouns and reflexives, the influence of gender marking is not entirely clear without a similar test in a language without any gender marking (e.g., Turkish).

Finally, one factor that might have contributed to the difference in accuracy between (12) and (15) is the different NP types of the target and intervener: names in (12), and full NPs in (15). Since reading times for the NPs alone were not reliably reported in previous studies on similarity-based interference in adults, the role of NP type in Experiments 1 and 2 is not apparent. These questions will be pursued in future research.

## Conclusion

The research presented in this paper has investigated children’s acquisition of adjunct control, using children’s non-adultlike behavior to test the predictions of a similarity-based interference account. While children’s grammars appear to be adultlike by age 4, we saw that their errors persist depending on the feature overlap between the grammatically accessible antecedent and a grammatically inaccessible intervener. As interference type effects are also observed in a number of other structures, both in children (realized as differences in accuracy) and in adults (as differences in reading times), these effects may account for children’s difficulties on a much more general scale, and point to a continuous developmental trajectory as children’s processing mechanisms become more resistant to interference.

## Author Contributions

JG and JL designed the study and materials. SZ and MP developed and adapted the task. JG ran the experiments, analyzed the data, and wrote a first draft of the paper. All authors worked on refining and revising the paper. All authors approved the final version.

## Conflict of Interest Statement

The authors declare that the research was conducted in the absence of any commercial or financial relationships that could be construed as a potential conflict of interest.

## Footnotes

1 The order used for introducing the characters in the description (10) was counterbalanced across items and lists, as was the order in the preamble (11) for the conjoined phrase.

2 With high accuracy observed especially in the MISMATCH conditions, the results also suggest that children’s grammars are adultlike. Children exhibited higher proportions of subject interpretations with less interference, which is not predicted unless there is at least a *preference* for the subject as the antecedent of PRO. This is consistent with a strict subject (adult) grammar, but not with a nominal or misattachment grammar. Furthermore, if children had a non-adult grammar with only a preference for the subject as antecedent of PRO (rather than a strict subject restriction), this would raise questions about the kind of evidence that children would need to transition to a strict subject grammar.
